# Factors affecting nurses’ acceptance of a digital triage platform in primary health care in Sweden: an extended UTAUT analysis

**DOI:** 10.1080/02813432.2026.2676776

**Published:** 2026-05-25

**Authors:** Rasmus Hermansson-Borrebaeck, Veronica Milos-Nymberg, Hanna Glock, Ulf Jakobsson, Patrik Midlöv, Susanna Calling

**Affiliations:** ^a^ Center for Primary Health Care Research, Department of Clinical Sciences Malmö, Lund University, Malmö, Sweden; ^b^ KRY Primary care, Region Skåne, Sweden; ^c^ University Clinic Primary Care, Skåne University Hospital, Region Skåne, Sweden

**Keywords:** eHealth, digital triage, technology acceptance, primary health care, UTAUT

## Abstract

**Background:**

Digital symptom checkers might help facilitate effective triage in primary healthcare. However, evidence regarding their acceptance among healthcare professionals remains limited. Identifying key factors of adoption is essential for successful integration into clinical practice.

This study aims to identify factors most relevant to nurses’ acceptance of a newly implemented digital triage platform and map their opinions regarding its use in Swedish primary healthcare.

**Method:**

A cross-sectional quantitative survey study based on an extended Unified Theory of Acceptance and Use of Technology (UTAUT) framework was distributed to nurses involved in digital triage at 82 primary healthcare centers in Sweden. 164 registered nurses participated. The platform, 1177-direct, is an online symptom checker used in triage. Associations between extended UTAUT constructs and ‘Intention to use/recommend’ were analyzed using linear regression models.

**Results:**

Nurses reported overall negative perceptions of the platform, with 61.6% expressing low ‘Intention to use/recommend’. All constructs showed significant associations with ‘Intention to use/recommend’ in simple regressions. In the multiple regression models, ‘Performance’ was the strongest construct (β = 0.75 and 0.58), followed by ‘Effort’ in the original UTAUT model and ‘Information transfer’ and ‘Work environment’ in the extended model.

**Conclusion:**

Nurses’ acceptance of the digital triage platform was primarily associated with perceived improvements in work performance. While extending the UTAUT model with context-specific constructs enhanced explanatory power, results should be interpreted with caution given limitations such as cross-sectional design, construct overlap and shared method variance. The results emphasize the need for contextualized frameworks when evaluating eHealth implementation in primary healthcare.

## Introduction

The benefits of eHealth are increasingly recognized across healthcare systems. Digital health technologies have demonstrated potential to enhance care quality, efficiency, and patient engagement while reducing costs and disparities in access to care (1–5).

Digital symptom checkers designed to facilitate triage represent one promising eHealth innovation. However, evidence supporting their effectiveness remains limited, and implementation often progresses faster than the corresponding research (6,7). Existing studies indicate that the accuracy of triage advice provided by these tools is generally low and tends to be risk-averse, frequently encouraging patients to seek professional care even for conditions suitable for self-care (8).

The majority of healthcare encounters occur in primary health care, therefore the need for an efficient and well-functioning triage system in this setting is substantial. In the Swedish healthcare system, approximately 60% of all in-person consultations take place in primary care, and in-person consultations represents only about 70% of its total encounters, as more than 30% are conducted remotely (9).

Successful integration of eHealth tools into clinical practice is crucial to realizing their potential benefits. Nevertheless, clinical implementation frequently falls short of expectations due to the inherent complexity of healthcare systems (10). Understanding barriers and facilitators to the adoption of eHealth tools is therefore essential for improving implementation outcomes.

The Unified Theory of Acceptance and Use of Technology (UTAUT) model was developed in 2003 by Venkatesh et al. It integrates key factors from eight earlier models of technology acceptance to provide a more comprehensive understanding of behavioral intention to use new technology and actual use behavior. One of its key advantages is its strong explanatory power. In prior studies UTAUT has accounted for approximately 70% of the variance in behavioral intention to use new technology, outperforming the earlier models (11).

Although some research has explored healthcare professionals’ perspectives on adopting eHealth technologies in general (12–14), studies conducted within the Swedish context have predominantly employed qualitative approaches (15–18). Quantitative studies conducted within primary healthcare remain scarce, particularly those focusing specifically on adoption of digital triage.

## Objective

To identify the factors most relevant to nurses’ acceptance of a newly implemented digital triage platform, using an extended UTAUT framework, and to map their opinions regarding the platform in clinical practice.

## Method

We developed, tested, and conducted a quantitative survey analysis based on the UTAUT framework. The survey was distributed to managers for all primary health care centers (PHCCs) in Southern Sweden (Region Skåne) that utilize the 1177-direct system (82 PHCCs), with instructions for redistribution to nurses involved in triage. The study was conducted between November 2024 and January 2025. An email was sent to all managers prior to the study deadline to remind them to redistribute the questionnaire to eligible nurses at their respective PHCCs. Participants were given the option to complete either a paper version of the questionnaire or a digital version administered through REDCap electronic case report forms (eCRF) (19,20). The two versions of the questionnaire may have exhibited minor differences in layout but were identical in content. Data were collected and subsequently analyzed from 164 registered nurses who responded to the survey.

## The platform

The digital platform in this study is 1177-direct, a patient-facing online symptom checker used in the triage process of primary healthcare. The platform is currently implemented in eleven of Sweden’s 21 healthcare regions (21). The platform employs an algorithm-based symptom checker that conducts the initial assessment using patient self-reported symptoms. Based on this assessment, patients may receive (I) automated self-care advice for non-complicated health issues, (II) referral to digital assessment by a nurse, or (III) a recommendation to attend an emergency department.

In a study with fictive cases based on real world cases that had previously been erroneously triaged *via* telephone triage the platform showed high levels of triage accuracy (91%) and safety (94%) when assessed against the Swedish National Triage Guidelines (22).

Digital nurse assessments are initiated through asynchronous chat and can, if deemed necessary by the nurse, be escalated to a videoconference. In most cases, the assessment is conducted by a nurse affiliated with the patient’s registered PHCC. Following the digital consultation, the nurse may schedule a physical appointment with either a nurse or a physician when required.

### UTAUT questionnaire

The UTAUT model (11) served as the primary framework for the questionnaire and was treated as a structured measurement instrument intended to capture theoretically defined constructs related to technology acceptance. At the same time, the instrument was extended (11,13,23) to fit the specific context of Swedish healthcare and the digital tool 1177-direct. This approach aimed to balance comparability with prior studies and contextual relevance.

To our knowledge, a validated Swedish version of the UTAUT questionnaire does not exist. Consequently, the questionnaire was translated into Swedish and subsequently retranslated by a native speaker to ensure accuracy. The UTAUT questionnaire contains 19 items. Sixteen of these items are grouped into four constructs: ‘performance expectancy’, ‘effort expectancy’, ‘social influence’, and ‘facilitating conditions’ (11). These constructs are theorized to predict ‘behavioral intention to use’ new technology, which represents the fifth construct and is measured by the remaining three items. By extension, behavioral intention to use is expected to predict actual use behavior. Interactions between the constructs and ‘intention to use’ are moderated by sex, age, experience, and voluntariness of use, as proposed in the original UTAUT framework. In an international context the UTAUT model is widely applied and validated (11).

For the purposes of this study, the original UTAUT questionnaire was modified. One item within the performance expectancy construct was removed (‘If I use the system, I will increase my chances of getting a raise’). In the facilitating conditions construct, one item (‘The system is not compatible with other systems I use’.) was adapted by removing the word ‘not’. In addition, the fifth construct, originally labeled ‘Behavioral intention to use the system’, was redefined as ‘Intention to use/recommend’. As the platform is already implemented and functions as an established entry point into the healthcare system, nurses are required to manage patients entering through this channel. Consequently, ‘Intention to use’ alone may partly reflect mandatory rather than voluntary use. To better capture participants’ willingness to engage with the system, the construct was expanded to include ‘Intention to recommend’. The three original items were reworked accordingly, and a new item was added.

**Figure 1.**
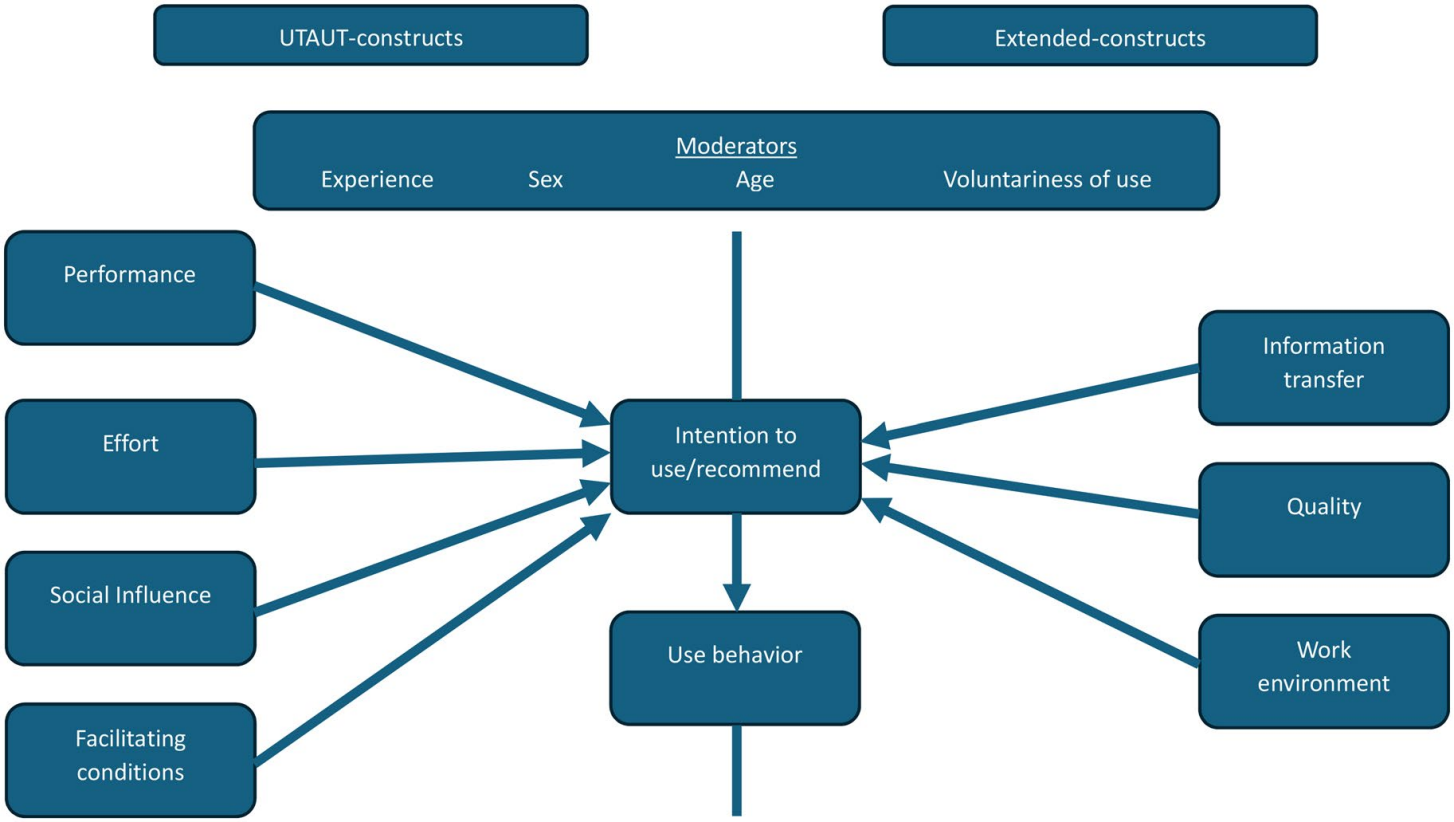
. **Research model:** This is a proposed extension of the Unified Theory of Acceptance and Use of Technology (UTAUT). Factors on the left side of the vertical line represent elements that, according to UTAUT, influence actual usage behavior. Factors on the right side are those we hypothesize may impact behavioral intention within the specific context of this study.

We also extended the UTAUT model to capture areas specific to the goals of the Swedish healthcare system and the 1177-direct platform. This extension included the construction of new items, which were grouped into three additional constructs: ‘information transfer’ (4 items), ‘quality’ (4 items), and ‘work environment’ (4 items). These constructs were intended to reflect aspects of importance to nurses working in triage, such as information transfer, perceived working conditions, and perceived triage quality, aspects that are not fully encompassed within the original UTAUT framework. The extended research model and a short explanation of the constructs can be found in Figure 1 and Table 1. A further set of items was included, which did not align precisely with the predefined constructs but were considered relevant to the aims of the study.

**Table 1.** Study constructs, construct descriptions and examples of questionnaire items.

Construct	Description	Example of item
Performance	The degree to which an individual believes that using the tool will help him or her to attain gains in job performance.	‘Using 1177-direct increases my productivity’
Effort	The degree of ease associated with the use of the tool.	‘1177-direct is easy to use’
Social influence	The degree to which an individual perceives that important others believe he or she should use the new tool.	‘People who influence my behavior think that I should use 1177-direct’
Facilitating conditions	The degree to which an individual believes that an organizational and technical infrastructure exists to support use of the tool.	‘I have the resources necessary to use 1177-direct’
Intention to use/recommend	The degree to which an individual intends to use or recommend the tool.	‘I want to continue using 1177-direct’ or ‘I would recommend that other units implement 1177-direct’
Information transfer	The degree to which an individual perceives that the tool improves information transfer from patient to healthcare professionals.	‘When triaging with 1177-direct, the ability to access relevant information is good’
Quality	The degree to which an individual perceives that the functions of the tool is of high quality.	‘1177-direct triages patients to an appropriate urgency/priority level’
Work environment	The degree to which an individual perceives that using the tool will affect job satisfaction	‘Satisfaction with the triage assignment is good when I work with 1177-direct’

The questionnaire underwent face-validity assessment, and the final version is presented in Appendix 1.

### Data analysis

The questionnaire employed 36 items rated on a 7-point Likert scale. Responses were recoded numerically and rescaled to a 0–100 range using min–max normalization at the item level. Standardized indices for each construct were then calculated as the mean of the corresponding items. For presentation purposes, item ratings and indexes were categorized as negative (rating 1–3, index 0–33), neutral (rating 4, index 34–66), and positive (rating 5–7, index 67–100).

Missing data were handled at the construct level. Because each construct comprised 3–4 items, any missing response represented a substantial proportion of the construct score. Cases with incomplete construct data were therefore excluded from analyses for that construct to avoid disproportionate item influence. The extent of missing data is reported in Table 4.

To assess the psychometric properties of the extended questionnaire, an exploratory factor analysis (EFA) was conducted for the newly developed constructs (information transfer, quality, and work environment). Principal component analysis with varimax rotation was applied. Sampling adequacy was evaluated using the Kaiser–Meyer–Olkin (KMO) measure and Bartlett’s test of sphericity.

Internal consistency was assessed using Cronbach’s alpha.

Construct validity was explored based on the factor structure and through analyses of convergent and discriminant validity. Convergent validity was examined using correlations between factor scores and corresponding construct indices (mean item scores), with correlations ≥ 0.50 considered acceptable. Discriminant validity was explored by the separation of constructs in the factor analysis and by inter-construct correlations, where values ≥ 0.70 indicated potential overlap. Factor loadings ≥ 0.40 were considered meaningful; cross-loadings were defined as loadings ≥ 0.40 on more than one factor with a difference of < 0.20, and loadings between 0.25 and 0.40 were considered secondary loadings.

Parametric statistical methods were employed, as they are widely regarded as robust for analyzing Likert-type data, even when the assumptions of normality or equal variance are not fully met (24,25). Statistical significance was defined at a threshold of *p* = 0.05. Relationships among components of the extended UTAUT model (Figure 1) were examined using bivariate Pearson correlation, interaction analyses, and linear regression. Variables identified through these analyses were subsequently included in multiple linear regression models and relevant subsets. All regression models were evaluated for multicollinearity, and model assumptions were verified through inspection of residuals using normal probability plots and scatter plots.

Statistical analyses were made with IBM SPSS Statistics for Windows, Version 29.0. Released 2022. Armonk, NY: IBM Corp.

### Ethical considerations

The project has been granted ethical approval from the Swedish Ethical Review Authority Dnr 2022-03883-01. A cover letter accompanied all questionnaires, providing written information about the study, data handling, and the voluntary nature of participation. Participants were informed in writing that submission of a completed questionnaire would be regarded as providing informed consent.

## Results

Table 2 presents participant characteristics. Data was collected from 164 registered nurses working with triage through the digital platform 1177-direct.

**Table 2.** Participant characteristics. *N* = 164.

Characteristic	Participants, n (%)
**Age (years)** ^a^	
<30	6 (3.7)
30-39	57 (34.8)
40-59	92 (56.1)
≥60	9 (5.5)
**Gender** ^b^	
Female	151 (92.1)
**Years in profession** ^a^	
<10	39 (23.8)
10-19	64 (39.0)
≥20	61 (37.2)
**Started using 1177-direct** ^c^	
>3 months ago	155 (94.5)
**Average use of 1177-direct in the past 3 months (visits/week)** ^a^	
0	2 (1.2)
1-10	143 (87.2)
11-20	15 (9.1)
>20	4 (2.4)

^a^Missing *n* = 0 (0%).

^b^Missing *n* = 4 (2.4%).

^c^Missing *n* = 2 (1.2%).

No statistically significant differences were observed between responses collected *via* paper-based and digital questionnaires across any of the constructs in independent samples t-test (‘Performance’ *p* = 0.573, ‘Effort’ *p* = 0.279, ‘Social influence’ *p* = 0.147, ‘Facilitating conditions’ *p* = 0.541, ‘Information transfer’ *p* = 0.522, ‘Quality’ *p* = 0.296, ‘Work environment’ *p* = 0.928, and ‘Intention to use/recommend’ *p* = 0.563), indicating no evidence of mode-related response bias.

Table 3 presents the results of an exploratory factor analysis, which demonstrated a Kaiser–Meyer–Olkin value of 0.876 and a significant Bartlett’s test of sphericity (*p* < 0.001), indicating suitability for factor analysis. The analysis yielded a three-component solution. Items related to ‘Work environment’ were mainly associated with a single component. Items reflecting ‘Information transfer’ also clustered largely on one component, whereas items related to ‘Quality’ were distributed across two components. Two items met the criteria for cross-loading, and two items showed secondary loadings. Correlations between construct indices and corresponding factor scores were high for ‘Information transfer’ (*r* = 0.916, *p* < 0.001) and ‘Work environment’ (*r* = 0.912, *p* < 0.001), and moderate for ‘Quality’ (*r* = 0.632, *p* < 0.001). Correlations between construct indices ranged from *r* = 0.441 to *r* = 0.757, with one correlation, between ‘Information transfer’ and ‘Quality’ (*r* = 0.757), exceeding the predefined threshold (≥ 0.70) for potential construct overlap.

**Table 3.** Rotated factor loadings from an exploratory factor analysis with varimax rotation.

	Factor 1	Factor 2	Factor 3
1177-direct triages patients to an appropriate urgency/priority level (‘Quality’)	0.869		
When triaging with 1177-direct, the ability to access relevant information is good (‘Information transfer’)	0.837		
The medical history report presented by 1177-direct is adequate (‘Information transfer’)	0.831		
Alarm symptoms in triage can be adequately detected when working with 1177-direct (‘Information transfer’)	0.828		
1177-direct triages some patients to self-care advice and others directly to ambulance. The patients triaged to contact with a nurse have been adequately triaged (‘Quality’)	0.779		
Patients’ expectations can be adequately captured during triage when using 1177-direct (Information transfer’)	0.644		0.500
I am allocated sufficient time to work with 1177-direct (‘Work environment’)		0.886	
I do not become stressed when working with 1177-direct (‘Work environment’)		0.780	
The administrative burden associated with triage is manageable when I work with 1177-direct (‘Work environment’)	0.316	0.715	0.352
Opportunities for joint assessment of triage cases with colleagues or members of other healthcare professions are good when working with 1177-direct (‘Quality’)			0.826
Overall, the quality of patient contact during triage is good when I work with 1177-direct (‘Quality’)	0.378		0.728
Satisfaction with the triage assignment is good when I work with 1177-direct (‘Work environment’)	0.433	0.537	0.541

Kaiser-Meyer-Olkin 0.876.

Bartlett’s test of sphericity *p* < 0.001.

Table 4 presents participants’ evaluations of the platform and the internal consistency of each construct. Cronbach’s alpha values indicated a high level of internal consistency across all constructs.

**Table 4.** Presentation of participants opinions. *n* = 164.

	Values				
Variables	Negative, n (%)	Neutral, n (%)	Positive, n (%)	Missing, n (%)	Cronbach α
**Separate items** ^a^ **^, b^**					
‘I received sufficient training before I started working with 1177-direct’	56 (34.1)	27 (16.5)	81 (49.4)	0 (0.0)	
‘I have been informed about the goal of implementing 1177-direct and why this goal is important’	44 (26.8)	32 (19.5)	87 (53.0)	1 (0.6)	
‘I can choose whether or not to work with1177-direct’	137 (83.5)	13 (7.9)	14 (8.5)	0 (0.0)	
‘The implementation of 1177-direct has benefited our patients’	70 (42.7)	43 (26.2)	51 (31.1)	0 (0.0)	
‘The implementation of 1177-direct has benefited our healthcare staff’	120 (73.2)	20 (12.2)	24 (14.6)	0 (0.0)	
**Constructs** ^c^					
Performance	103 (62.8)	43 (26.2)	15 (9.1)	3 (1.8)	0.88
Effort	31 (18.9)	61 (37.2)	69 (42.1)	3 (1.8)	0.90
Social influence	25 (15.2)	86 (52.4)	51 (31.1)	2 (1.2)	0.63
Facilitating conditions	44 (26.8)	79 (48.2)	37 (22.6)	4 (2.4)	0.69
Intention to use/recommend	101 (61.6)	32 (19.5)	29 (17.7)	2 (1.2)	0.97
Information transfer	71 (43.3)	68 (41.5)	23 (14.0)	2 (1.2)	0.89
Quality	51 (31.1)	80 (48.8)	28 (17.1)	5 (3.0)	0.75
Work environment	84 (51.2)	58 (35.4)	21 (12.8)	1 (0.6)	0.82

^a^7 point Likert scale: Negative = 1-3, Neutral = 4, Positive = 5-7.

^b^Items have been translated to English for presentational purposes (Swedish in the questionnaire).

^c^0-100 index scale: Negative = 0-33.3, Neutral = 33.4-66.5, Positive = 66.6-100.

Overall, participants reported neutral to negative perceptions regarding whether the introduction of the platform had benefited patients, and predominantly negative perceptions regarding its benefits for healthcare professionals. Ratings were negative for ‘Intention to use/recommend’, ‘Performance’, and ‘Work environment’; neutral to negative for ‘Information transfer’ and ‘Quality’; neutral for ‘Facilitating conditions’; and neutral to positive for ‘Effort’ and ‘Social influence’. Furthermore, 84% of participants reported negative responses regarding ‘Voluntariness of use’.

Table 5 presents results from simple linear regression analyses. All constructs, including both the original UTAUT constructs and the additional constructs developed specifically for this study, were significantly associated with ‘Intention to use/recommend’ (*R^2^* = 0.17–0.74, *p* < 0.001).

**Table 5.** Simple linear regression.

Predictor	R^2^	B-level (95% CI)	p-value
UTAUT			
Performance	0.74	1.16 (1.05-1.27)	<0.001
Effort	0.31	0.75 (0.57-0.92)	<0.001
Social Influence	0.17	0.66 (0.43-0.88)	<0.001
Facilitating Conditions	0.24	0.73 (0.52-0.93)	<0.001
Specific			
Information Transfer	0.43	0.88 (0.72-1.04)	<0.001
Quality	0.44	1.03 (0.85-1.22)	<0.001
Work Environment	0.46	0.85 (0.71-1.00)	<0.001

Analysis of the proposed moderators within the UTAUT framework identified ‘Experience in profession’ and ‘Voluntariness of use’ as confounding variables, both demonstrating significant correlations with ‘Intention to use/recommend’ and at least one construct, and influencing a change in unstandardized *B* greater than 10% in stepwise linear regression analysis. ‘Voluntariness of use’ was the only potential moderator showing a significant interaction effect (‘Voluntariness of use × Social influence’ *B* = 0.166 (95% CI 0.029-0.302), *p* = 0.018). The confounders and interaction terms were included in the final regression models.

Table 6 presents the two final multiple linear regression models.

**Table 6.** Multiple linear regression.

	UTAUT	Extended UTAUT
**Model statistics**		
Adjusted R^2^	0.772	0.815
P-value	<0.001	<0.001
VIF-value range	1.096 − 2.155	1.093 − 3.140
**Independent variables – constructs**		
**Performance**		
Β-level (95% CI)	1.017 (0.892-1.142)	0.790 (0.649-0.931)
Β-level ‘Standardized’	0.752	0.582
P-value	<0.001	<0.001
**Effort**		
Β-level (95% CI)	0.183 (0.036-0.330)	0.129 (-0.015-0.274)
Β-level ‘Standardized’	0.135	0.093
P-value	0.015	0.079
**Social influence**		
Β-level (95% CI)	−0.005 (-0.158-0.148)	−0.032 (-0.173-0.108)
Β-level ‘Standardized’	−0.003	−0.020
P-value	0.950	0.651
**Facilitating conditions**		
Β-level (95% CI)	0.046 (-0.123-0.216)	−0.088 (-0.253-0.078)
Β-level ‘Standardized’	0.031	−0.058
P-value	0.591	0.296
**Information transfer**		
Β-level (95% CI)		0.273 (0.121-0.426)
Β-level ‘Standardized’		0.202
P-value		<0.001
**Quality**		
Β-level (95% CI)		0.036 (-0.159-0.231)
Β-level ‘Standardized’		0.023
P-value		0.718
**Work environment**		
Β-level (95% CI)		0.234 (0.108-0.360)
Β-level ‘Standardized’		0.184
P-value		<0.001
**Independent variables – Moderators/Confounders and Interaction terms**		
**Voluntariness of use**		
Β-level (95% CI)	0.739 (-0.917-2.395)	0.284 (-1.251-1.820)
Β-level ‘Standardized’	0.037	0.014
P-value	0.379	0.715
**Experience in profession**		
Β-level (95% CI)	−3.312 (-6.582–0.042)	−3.811(-6.845–0.778)
Β-level ‘Standardized’	−0.081	−0.093
P-value	0.047	0.014
**Social Influence X Voluntariness of use (centered)**		
Β-level (95% CI)	0.058 (-0.020-0.135)	0.056 (-0.015-0.126)
Β-level ‘Standardized’	0.060	0.058
P-value	0.143	0.121

The first model included the original UTAUT constructs, along with the identified confounders and interaction terms (adjusted R^2^ = 0.77). Significant associations were observed for ‘Performance’ (*p* < 0.001) and ‘Effort’ (*p* = 0.015).

The second model also incorporated the newly developed constructs, yielding an improved fit (adjusted R^2^ = 0.82). Significant associations were observed for ‘Performance’ (*p* < 0.001), ‘Information transfer’ (*p* < 0.001), and ‘Work environment’ (*p* < 0.001).

The confounder ‘Experience in profession’ was significantly associated with the outcome in both models (*p* = 0.047 and *p* = 0.014, respectively). Among all predictors, ‘Performance’ accounted for the largest proportion of variance in ‘Intention to use/recommend’ (Standardized β = 0.75 and 0.58, respectively). No evidence of problematic multicollinearity was found, with variance inflation factor (VIF) values ranging from 1.1 to 2.2 in the first model and 1.1 to 3.1 in the second. The interaction term (‘Social influence × Voluntariness of use’) was mean-centered to reduce multicollinearity.

To explore potential age-related differences, the sample was divided into two groups: ≤39 years (*n* = 63, 38.4%) and ≥40 years (*n* = 101, 61.6%). In the final model including all constructs, confounders, and the interaction terms, significant associations were observed between ‘Intention to use/recommend’ and ‘Performance’ (*B* = 0.814 (95% CI 0.586 − 1.042), *p* < 0.001), ‘Effort’ (*B* = 0.284 (95% CI 0.026 − 0.543), *p* = 0.031), and ‘Work environment’ (*B* = 0.272 (95% CI 0.024 − 0.520), *p* = 0.032) for the younger group. For the older group, significant associations were observed between ‘Intention to use/recommend’ and ‘Performance’ (*B* = 0.797 (95% CI 0.600 − 0.994), *p* < 0.001), ‘Information transfer’ (*B* = 0.222 (95% CI 0.019 − 0.425), *p* = 0.033), and ‘Work environment’ (*B* = 0.241 (95% CI 0.078 − 0.403), *p* = 0.004).

## Discussion

This study explored the factors that most strongly influenced nurses’ acceptance of a new digital triage platform in the Swedish primary healthcare setting. Overall opinions of the platform were unfavorable, 61.6% of the nurses reported a negative index in the construct ‘intention to use/recommend’. An extended UTAUT model was applied. All constructs, both original and those developed for this study, showed significant associations with the outcome variable, ‘intention to use/recommend’, in linear regression analyses.

Two multiple linear regression models were assessed. In both models, the construct ‘performance’ remained significantly associated with the outcome, and accounted for the largest proportion of the variance (75% in the original UTAUT model and 58% in the extended model). These findings suggest that the perceived ability of the tool to improve work performance was the most influential factor, surpassing considerations related to areas such as improved work environment and perceived quality of the tool.

Previous research on the adoption of eHealth technologies among healthcare professionals shows varying results regarding the key determinants of acceptance. These results appear to be shaped by, and dependent on, the healthcare system and the specific context in which the eHealth tools are introduced.

Similar to our results, in a study set in the primary health care setting in Finland, Kujala et al. found that ‘benefits to professionals’ work’ were the strongest predictors of support for web-based symptom checkers among healthcare professionals (26). In contrast, a systematic review by Wang et al. investigating eHealth adoption in low- and middle-income countries identified ‘facilitating conditions’ as the most influential factor (14). Nevertheless, their review also highlighted that concerns related to ‘performance expectancy’ and ‘effort expectancy’ were key barriers to adoption. Furthermore, a study from China by Dai et al. examining AI-based digital health tools found ‘effort expectancy’ to be the dominant predictor of intention to use (27).

Subgroup analyses in previous work have also revealed nuanced effects. For example, Barchielli et al. reported that ‘social influence’ played a greater role among younger and less experienced nurses (28). In contrast, in our study, ‘social influence’ was not significantly associated with ‘intention to use/recommend’ in any age group in the final regression models. However, we observed that ‘effort expectancy’ was a significant factor in the younger group, while ‘information transfer’ was significant among older participants.

These comparisons emphasize how contextual and demographic factors shape the relevance of specific constructs and underline the value of adapting theoretical models to the healthcare environment under study.

## Limitations and interpretative concerns

Comparisons between studies are inherently limited by differences in study design, context, and the theoretical frameworks employed. While some investigations draw upon the UTAUT model, the use of varying extended versions complicates direct comparison. This study employed an extended UTAUT framework, building on previous research such as Li et al., who argued that the original model, though valuable, may be too narrow to fully capture the complexity of the eHealth technology adoption process (13).

Expanding the model with additional constructs yielded a more comprehensive understanding of the determinants of acceptance, which is a strength of this study. All added constructs demonstrated significant associations with ‘intention to use/recommend’, and the extended model accounted for a higher proportion of explained variance than the original version. However, this increased explanatory power may partly reflect conceptual overlap between constructs and model specification rather than independent explanatory contributions, and may also indicate a degree of model overfitting. This enhanced explanatory power also comes at the expense of comparability with other studies, a limitation that underscores the ongoing need for harmonized frameworks in eHealth acceptance research. Building on these methodological insights, future work may benefit from testing and refining extended acceptance models across different healthcare contexts to improve both explanatory power and cross-study comparability.

Furthermore, the observed associations in the study should be interpreted with caution. As the data are cross-sectional and based on self-reported measures in a single survey instrument, causal inferences cannot be drawn, and potential reverse causal relationships cannot be ruled out. Shared method variance may also have influenced the results.

The methodology used for survey distribution, i.e. distribution to nurses involved in digital triage *via* managers for the PHCCs, means that definitive data on the response rate are not available. This introduces a potential risk of selection and non-response bias, as it is unclear to what extent the respondents are representative of the full population. However, we received 164 responses from 82 PHCCs suggesting the response rate was acceptable. Furthermore, the large number of PHCCs included, both rural and urban and across a wide range of socioeconomic areas, may support generalizability of the findings.

For descriptive purposes, index values were categorized into ‘negative’, ‘neutral’, and ‘positive’, which may simplify the underlying continuous response distributions.

Although the results provide some preliminary support for the reliability and convergent construct validity of the modified instrument, cross-loadings, inter-construct correlations, and the tendency for items related to ‘Information transfer’ and ‘Quality’ to load on the same component indicate limited discriminant validity between some constructs. In addition, the absence of confirmatory factor analysis and more advanced psychometric validation methods represents a limitation. The extended constructs should therefore be interpreted as exploratory and require further validation in future studies.

## Conclusion

Nurses’ acceptance of the 1177-direct digital triage platform was most strongly associated with perceived improvements in work performance. These results might add insight when developing and implementing new digital platforms. Extending the UTAUT model with additional constructs such as ‘quality’, ‘work environment’ and ‘information transfer’ enhanced the explanatory power in this study, but these findings should be interpreted with caution due to key limitations such as a cross-sectional design and potential conceptual overlap between the constructs in the extended model. Nevertheless, the results highlight the importance of a contextualized framework when studying eHealth adoption in healthcare settings.

Further research is needed to validate the extended model and to assess its applicability in other healthcare settings and populations.

## Supplementary Material

Appendix 1 Survey 1177 direct.docx

## Data Availability

The datasets generated and analyzed during the study are available from the corresponding author upon reasonable request. However, due to ethical considerations, the data are not publicly accessible.
