# Supplementary material for: Factors affecting nurses’ acceptance of a digital triage platform in primary health care in Sweden: an extended UTAUT analysis
Source: Scand J Prim Health Care. 2026 May 25;44(1):2676776. doi: 10.1080/02813432.2026.2676776 (PMC13202699; doi:10.1080/02813432.2026.2676776)
Supplement: Appendix 1 Survey 1177 direct.docx [file IPRI_A_2676776_SM7925.docx]

This is a survey study on healthcare professionals’ experiences of working with digital triage. The survey is part of a larger scientific evaluation of digital triage being conducted at the Centre for Primary Care Research. Currently, there is limited research on digital triage, so this study will provide new knowledge.

The survey mainly consists of various statements about digital triage. Next to each statement is a 7-point scale ranging from “Strongly disagree” to “Strongly agree” Please tick the box that best corresponds to your opinion. The estimated time to complete the survey is 5–10 minutes.

Your participation is voluntary, and your responses will be treated anonymously and confidentially. The study has been approved by the Swedish Ethical Review Authority (Dnr 2022-03883-01). Individual responses cannot be identified in the final report or in scientific publications. In accordance with the Data Protection Regulation (DSF) and the General Data Protection Regulation (GDPR), we only request the information necessary for our analysis. For questions specifically about DSF/GDPR, you can contact the Data Protection Officer at [dataskydd.pv@skane.se](mailto:dataskydd.pv@skane.se).

**By submitting the survey, you also consent to the processing of the information you have provided.**

If you have any questions, please feel free to contact us.

Thank you in advance for your time and engagement!

Kind regards,

Rasmus Hermansson-Borrebaeck Susanna Calling

PhD student, Specialist in General Practice Associate Professor, Specialist in General Practice

Center for Primary Health Care Research

Lund University

Part 1: Statements regarding the use of new technologies. Where applicable, please compare with a corresponding experience using telephone triage. For each statement, tick the box that best corresponds to your opinion.

|  | Strongly disagree | Disagree | Somewhat disagree | Neutral | Somewhat agree | Agree | Strongly agree |
| --- | --- | --- | --- | --- | --- | --- | --- |
| 1. 1177-direct is useful in my job |  |  |  |  |  |  |  |
| 1. Using 1177-direct enables me to accomplish tasks more quickly |  |  |  |  |  |  |  |
| 1. Using 1177-direct increases my productivity |  |  |  |  |  |  |  |
| 1. My interaction with 1177-direct is clear and understandable (refers to your interaction with the system, not the patient) |  |  |  |  |  |  |  |
| 1. It was easy for me to become skillful at using 1177-direct |  |  |  |  |  |  |  |
| 1. 1177-direct is easy to use |  |  |  |  |  |  |  |
| 1. Learning to operate 1177-direct was easy for me |  |  |  |  |  |  |  |
| 1. People who influence my behaviour (e.g. my immediate supervisor) think that I should use 1177-direct |  |  |  |  |  |  |  |
| 1. People who are important to me (e.g. my colleagues) think that I should use 1177-direct |  |  |  |  |  |  |  |

Part 1: Statements regarding the use of new technologies. Where applicable, please compare with a corresponding experience using telephone triage. For each statement, tick the box that best corresponds to your opinion.

|  | Strongly disagree | Disagree | Somewhat disagree | Neutral | Somewhat agree | Agree | Strongly agree |
| --- | --- | --- | --- | --- | --- | --- | --- |
| 1. The department manager has been helpful in the use of 1177-direct |  |  |  |  |  |  |  |
| 1. The organization (Region Skåne) has been helpful in the use of 1177-direct |  |  |  |  |  |  |  |
| 1. I have the resources necessary to use 1177-direct |  |  |  |  |  |  |  |
| 1. I have the knowledge necessary to use 1177-direct |  |  |  |  |  |  |  |
| 1. 1177-direct is compatible with other systems I use |  |  |  |  |  |  |  |
| 1. A specific person (or group) is available for assistance with system difficulties when using 1177-direct |  |  |  |  |  |  |  |
| 1. I want to continue using 1177-direct |  |  |  |  |  |  |  |
| 1. I am satisfied with 1177-direct |  |  |  |  |  |  |  |
| 1. I think that 1177-direct is a good complement to telephone triage |  |  |  |  |  |  |  |
| 1. I would recommend that other units implement 1177-direct |  |  |  |  |  |  |  |

Part 2: Additional statements regarding the 1177-direct tool. For each statement, tick the box that best corresponds to your opinion.

|  | Strongly disagree | Disagree | Somewhat disagree | Neutral | Somewhat agree | Agree | Strongly agree |
| --- | --- | --- | --- | --- | --- | --- | --- |
| 1. The medical history report presented by 1177-direct is adequate |  |  |  |  |  |  |  |
| 1. When triaging with 1177-direct, the ability to access relevant information is good. |  |  |  |  |  |  |  |
| 1. Alarm symptoms in triage can be adequately detected when working with 1177-direct. |  |  |  |  |  |  |  |
| 1. Patients’ expectations can be adequately captured during triage when using 1177-direct |  |  |  |  |  |  |  |
| 1. 1177-direct triages some patients to self-care advice and others directly to ambulance. The patients triaged to contact with a nurse have been adequately triaged |  |  |  |  |  |  |  |
| 1. 1177-direct triages patients to an appropriate urgency/priority level |  |  |  |  |  |  |  |
| 1. Overall, the quality of patient contact during triage is good when I work with 1177-direct. |  |  |  |  |  |  |  |
| 1. Opportunities for joint assessment of triage cases with colleagues or members of other healthcare professions are good when working with 1177-direct |  |  |  |  |  |  |  |

Part 2: Additional statements regarding the 1177-direct tool. For each statement, tick the box that best corresponds to your opinion.

|  | Strongly disagree | Disagree | Somewhat disagree | Neutral | Somewhat agree | Agree | Strongly agree |
| --- | --- | --- | --- | --- | --- | --- | --- |
| 1. The administrative burden associated with triage is manageable when I work with 1177-direct |  |  |  |  |  |  |  |
| 1. Satisfaction with the triage assignment is good when I work with 1177-direct |  |  |  |  |  |  |  |
| 1. I do not become stressed when working with 1177-direct |  |  |  |  |  |  |  |
| 1. I am allocated sufficient time to work with 1177-direct |  |  |  |  |  |  |  |
| 1. I received sufficient training before I started working with 1177-direct |  |  |  |  |  |  |  |
| 1. I have been informed about the goal of implementing 1177-direct and why this goal is important |  |  |  |  |  |  |  |
| 1. I can choose whether or not to work with 1177-direct |  |  |  |  |  |  |  |
| 1. The implementation of 1177-direct has benefited our patients |  |  |  |  |  |  |  |
| 1. The implementation of 1177-direct has benefited our healthcare staff |  |  |  |  |  |  |  |

Part 3: To best analyze, generalize, and use the results, we need some information about you. After that, there is space for open comments.

**37. Age (years):**
Under 30 years  30–39 years  40–59 years  60 years or older

**38. Legal sex:**
Male  Female

**39. Profession:**
Registered Nurse  Other – please specify: _______

**40. Years in the profession:**
Less than 10 years  10–19 years  20 years or more

**41. Current clinical employment percentage in primary care:**
50% or less  51–75%  76–100%

**42. I started working with the tool:**
Less than 1 month ago  1–3 months ago  More than 3 months ago

**43. Over the past 3 months, I have on average worked in the tool during:**
0 visits/week  1–10 visits/week  11–20 visits/week  >20 visits/week

**44. Additional comments?**
